# Numerical Optimization of a Microfluidic Assisted Microarray for the Detection of Biochemical Interactions

**DOI:** 10.3390/s111009658

**Published:** 2011-10-12

**Authors:** Emanuele Orabona, Ilaria Rea, Ivo Rendina, Luca De Stefano

**Affiliations:** 1 Institute for Microelectronics and Microsystems, Naples Unit, National Research Council, Via P. Castellino 111, 80131 Napoli, Italy; E-Mails: ilaria.rea@na.imm.cnr.it (I.R.); ivo.rendina@na.imm.cnr.it (I.R.); luca.destefano@na.imm.cnr.it (L.D.S.); 2 Department of Physics, University of Naples “Federico II”, Via Cinthia, 80128 Napoli, Italy

**Keywords:** microfluidics, biosensing, reaction kinetics

## Abstract

Finite element method analysis was applied to the characterization of the biomolecular interactions taking place in a microfluidic assisted microarray. Numerical simulations have been used for the optimization of geometrical and physical parameters of the sensing device. Different configurations have been analyzed and general considerations have been derived. We have shown that a parallel disposition of the sensing area allows the homogeneous formation of the target molecular complex in all the active zones of the microarray. Stationary and time dependent results have also been obtained.

## 1. Introduction

In the past two decades microfluidics has emerged as a powerful tool for biosensing [1] and biophotonics [2]. Microfluidic devices require small reagent volumes, short reaction times and allow high throughput due to their parallel mode of operation. Microfluidics represent a fundamental tool to integrate almost all the functionality of a laboratory onto a single chip, *i.e.*, a lab-on-a-chip. Microfluidics also hold promise for many other applications, such as the manipulation of nanomaterials [3,4]. In recent years, the study of microfluidic systems for biosensing has become an active research field. Biosensors exploit a variety of different detection mechanisms such as microcantilever based transducers [5], surface plasmon resonance sensors [6], and porous silicon based biosensors [7,8]. In these devices, the selectivity, *i.e.*, the ability to quantify a particular target analyte in a complex mixture, is due to some specific interaction between a bioprobe, such as a DNA single strand or a protein or an enzyme, and its own ligand. Even if the basic principles of molecular interaction detection are completely different, a common key issue is thus the analyte-ligand binding kinetics. The specific and selective recognition of analytes occurs at the reacting surface of the biosensor, which is a solid-liquid interface. The reaction kinetics can be described as a two-step process; namely, a mass-transport process, which takes into account the diffusion or the dragging of molecules in the fluids, and a chemical surface reaction process, which depends strictly on molecular interactions.

Many works concerning the modeling of a microfluidic biosensor have appeared recently. The main aim of these studies usually was to improve some aspect of the sensing performance, such as sensitivity, time response, and dependence on external factors. How the assay parameters determine the amount of captured analytes [9], the optimization of a microfluidic channel in case of a nanowire biosensor [10], the electro-thermal effect on diffusion enhancing [11], and a novel design for fiber-optic localized plasmon resonance biosensor [12] are among the topics that have been studied.

Among biosensors, the microarray technology has demonstrated a great potential in drug discovery, proteomics research, and medical diagnostics. The reason of this success is the very high throughput of these devices due to the large number of samples that can be analyzed simultaneously in a single parallel experiment. The microarray technology is based on the immobilization of a huge amount of bioprobes on a solid platform, which can be obtained by *in situ* direct synthesis of the biomolecules or by binding them on a functionalized area.

The convergence between microfluidics and microarrays has been relatively straightforward due to their multiple shared features, but the implementation of a microfluidic circuit on an array device is not trivial nor simple: a specific design is often required to meet biological constraints and fabrication technique demands. In this context, numerical simulations by finite element methods (FEM) allow a space and time characterization of the biomolecule distribution and interaction in the circuit. Hu *et al.* explained in [13] the different antigen-antibody binding kinetic between four sensing elements, proposing a “zigzag” array configuration to improve binding uniformity; Lee *et al.* proposed a recirculating flow system for a microfluidic DNA microarray to improve the rate of hybridization [14]; Srivannavit *et al.* instead proposed a microfluidic reactor array for massively parallel *in situ* synthesis of oligonucleotides obtaining a quite uniform binding kinetics on to the array [15].

In this work, we present a numerical study by FEM analysis of the binding interaction between active sites on the array surface elements with biochemical species in microfluidic networks. While the literature works generally consider interactions between biochemical species under flow conditions, in our simulation we have also considered the binding kinetics under static conditions, with an initial step involving flow of a liquid solution to fill the channel, followed by a flow velocity decreasing to a zero value, and we have compared the results with respect to the dynamic approach. Many experiments, especially those requiring consumption of a very low volume of reagent for economic or technical reasons, are driven in static, or quasi-static, steady flow conditions, so this is a useful design tool for both situations. On the basis of the results obtained, we also propose a new microfluidic layout for parallel flow to provide efficient and uniform analyte distribution on the sensing part of microfluidic assisted microarrays.

## 2. Theory

The modeling of what happens before transduction of a biomolecular interaction in a biosensor requires considering at least three physical processes: (1) the surface reactions, *i.e.*, the binding of a biomolecule onto the functionalized surface; (2) the fluid flow in microchannels, which takes into account the mass transport in the microfluidic circuit; (3) the diffusion of chemical species, which is the only process for bringing an analyte to the active site(s). The interaction [Equation (1)] between one chemical species A (mol/m^2^), bound to the sensing area, and a second chemical species B (mol/m^3^), present in a buffer solution, producing a complex C created by the two molecular species, can be described by the first order time-dependent Langmuir Equation [Equation (2)]:

(1) $$A+B\overset{k_{a}}{\underset{k_{d}}{⇄}}C$$

(2) $$\frac{\partial C}{\partial t}=k_{a}A\cdot (B-C)-k_{d}C$$

where C is measured in mol/m^2^, k_a_ is the association rate constant (M^−1^s^−1^), and k_d_ is the dissociation rate constant (s^−1^). This equation can be used for antigen-antibody [10,13] or protein-ligand reactions [11] or other biochemical interactions. The equilibrium complex concentration C_eq_ can be expressed as:

(3) $$C_{\mathrm{eq}}=\frac{\mathrm{AB}}{A+k_{d}/k_{a}}$$

where the ratio *k_d_/k_a_* represents the inverse of the affinity constant. The response of a biosensor is proportional to the amount of the compound C formed on the sensing regions. It is crucial to understand the behaviour of the complex formation rate C(t) and equilibrium concentration C_eq_ in the microfluidic configuration assigned in order to maximize the sensor response as a function of the fabrication parameters.

The fluid flow can be modelled using the Navier-Stokes equations with the incompressibility condition:

(4) $$\frac{\partial \vec{u}}{\partial t}+\vec{u}\cdot \nabla \vec{u}=-\frac{\nabla p}{\rho }+\frac{\mu }{\rho }\nabla^{2}\vec{u}$$

(5) $$\nabla \cdot \vec{u}=0$$

where u, p, ρ and μ are the velocity field, the pressure, the density and dynamic viscosity of the fluid, respectively. The values of the last two fluid constants are assumed as those of water: ρ = 10^3^ kg/m^3^ and μ = 10^−3^ Pa·s. The flow is considered laminar with a parabolic profile at the inlet and an average velocity u_0_, since the flow in the microchannel is in the low Reynolds number region. Boundary conditions for the equations are p = 0 at the outlet and no-slip walls (u = 0) elsewhere.

Moreover, the transport of the chemical species B in bulk liquid phase is described by the convection and diffusion equation:

(6) $$\frac{\partial B}{\partial t}+\vec{u}\cdot \nabla B=D\nabla^{2}B$$

where D (m^2^/s) is the diffusion coefficient of the chemical species B in bulk phase. Complete boundary conditions are the following:

(7) $$\begin{array}{cc} B=B_{0} & \text{at the inlet};\\ \vec{n}\cdot (D\nabla B)=0 & \text{at the outlet};\\ \vec{n}\cdot (c\vec{u}-D\nabla B)=0 & \text{at the microchannel walls};\\ \vec{n}\cdot (B\vec{u}-D\nabla B)=-\frac{\partial C}{\partial t} & \text{at the reaction surfaces.} \end{array}$$

where *n⃗* is the unit normal vector to the surface.

In order to simulate a static process, we have multiplied inlet conditions B_0_ and u_0_ for the function 1-H(t-t_fill_), where the H(t) is the Heaviside step function and t_fill_ is the necessary time to fill the channel given by L/u_0_ where L is the total device length. In this way we can simulate the injection of the solution of B for t_fill_ sec in the microchannel and then the subsequent static incubation. Under dynamic conditions a constant flow velocity in the microchannels is assumed. The numerical calculations have been performed using the FEMLAB™ (Comsol Inc.) finite element software package combining the three differential equations into a single model.

## 3. Numerical Simulations

**Figure 1.**
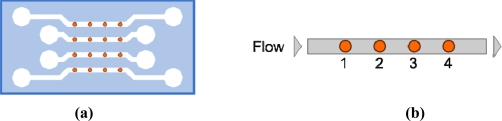
**(a)** An examplary scheme of the microfluidic assisted microarray, **(b)** scheme of the channel with four sensing elements used in our model.

The microarray that we have considered is composed by sixteen elements arranged in 4 × 4 matrix: each circular element has a radius of 100 μm and they are spaced 600 μm apart. In this work, we propose two different microfluidic configurations; an example of the first one is sketched in Figure 1(a). The microfluidic channels are 250 μm wide, 10 μm high, and 3 mm long and connect the elements as show in Figure 1(b). The distance between the inlet and the first element is the same as between the last element and outlet which has been considered equal to 300 μm. For our simulations, we have assumed typical value for k_a_ and k_d_ (k_a_ = 5 × 10^5^ M^−1^ s^−1^, k_d_ = 10^−4^s^−1^) [16], a typical diffusion coefficient for D = 10^−11^ m^2^/s and an active site surface concentration A = 1 × 10^−8^ mol/m^2^.

**Figure 2.**
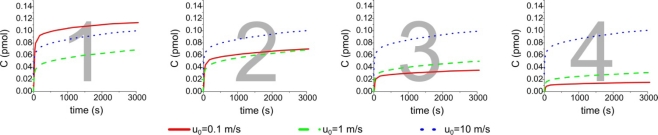
Comparison of the formation of complex C simulating a static incubation from the first to the last element in a linear microchannel for different inlet velocity values.

We have also assumed B_0_ = 10 nM and u_0_ ranging from 0.1 to 10 mm/s: we have studied the formation of compound C in the sensing region by changing the inlet velocity. We have chosen to change this parameter because it doesn’t directly influence the equilibrium complex concentration C_eq_ [Equation (3)] and can be easily controlled in real experiments by an automatic pump. Other parameters, such as the diffusion coefficient or the affinity constant, can be adapted according to the chemical species considered. The results of the amount of C on the surface of four elements under static steady flow conditions in a single channel are shown in Figure 2.

The simulation has shown that there is a clear decrease in the formation of compound C which is proportional to the inlet distance from the first to the fourth element, respectively. The effect is due to a concentration decrease along the channel of the chemical species B: by increasing the inlet velocity up to 10 mm/s more homogeneous values among the four active surfaces can be obtained. A change of the inlet position will cause only a time shift in the graphs of Figure 2.

**Figure 3.**
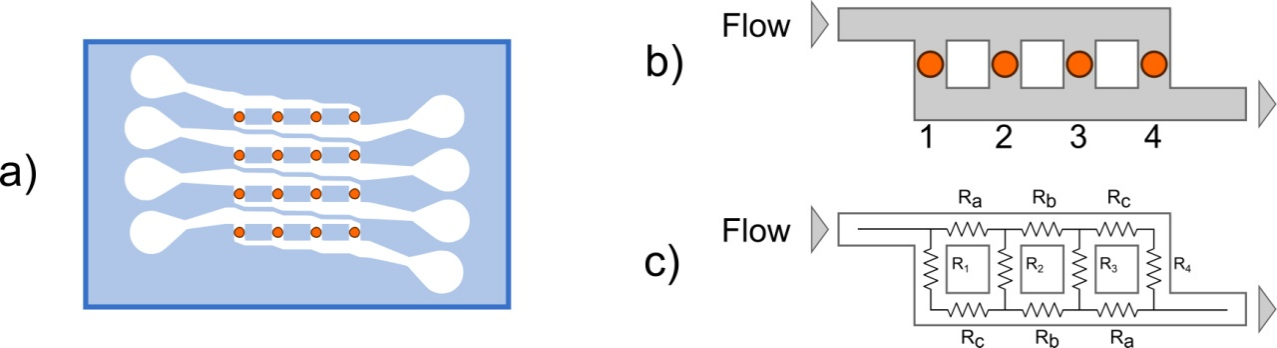
**(a)** An example scheme of the microarray with the new microfluidic layout proposed, **(b)** the scheme of the channel with four sensing elements used in our model, and **(c)** its electrical model.

The second layout that we propose is viewed as an improvement of the device using the same element configuration, but changing the microfluidic network. In this design, we propose a parallel approach [see Figure 3(a)] with four parallel channels which transport the chemical species, thus avoiding the formation of a different surface density of the compound C on the sensing elements.

**Figure 4.**
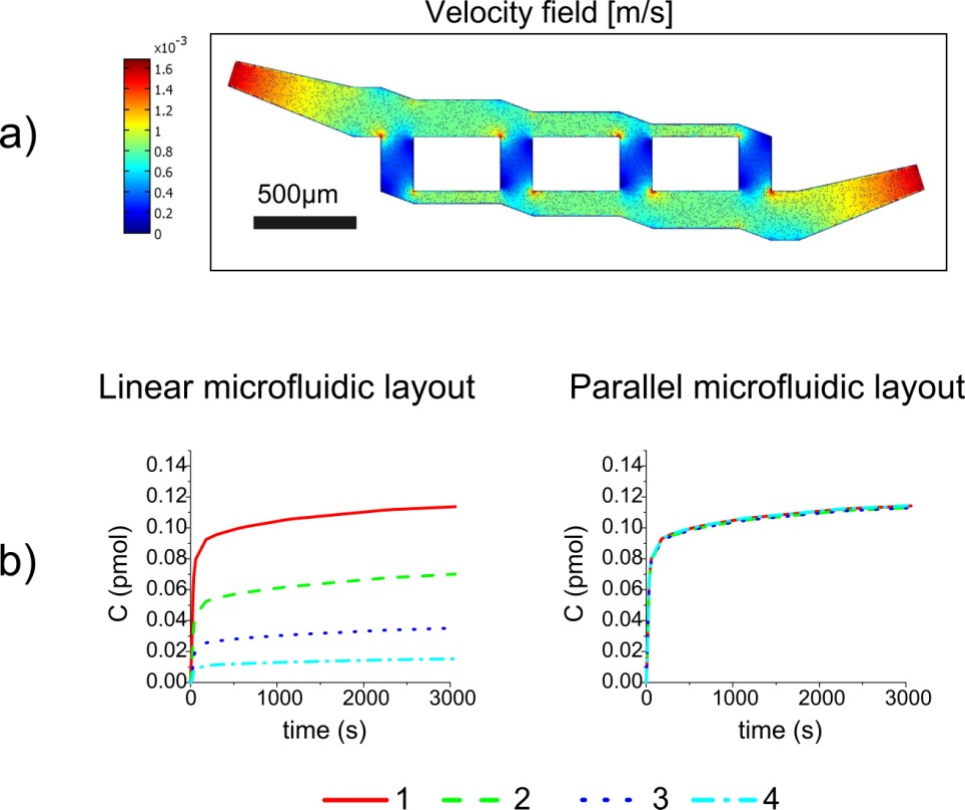
**(a)** Fluid dynamic simulation by FEMLAB™ of the new proposed microfluidic layout. **(b)** Comparison of binding kinetics obtained simulating a static incubation from the first to the last element in linear and the parallel microfluidic systems, respectively.

Since under static flow conditions, a homogeneous distribution of C along the channel depends on the average inlet velocity, if we want the same density of C in the parallel active sites, we must have the same local velocity. The pressure driven, steady-state flow of an incompressible fluid through a straight channel can be described by the Hagen-Poiseuille law:

(8) $$\Delta p=R_{\mathrm{hyd}}Q$$

where Δ*p* is the pressure difference along the channel, *R_hyd_* is the hydraulic resistance and *Q* is the flow rate, which is related to the local velocity. From a formal point of view, this law is completely analogous to the Ohm’s law Δ*V* = *RI*, which relates the electrical current I through a wire with the electrical resistance R and the electrical potential difference Δ*V* along the wire. Inspired by this analogy, the microfluidic network can be modelled as an electrical network [Figure 3(b)], and the hydraulic resistance of each channel can be calculated by the following equation [17]:

(9) $$R_{\mathrm{hyd}}=\frac{12\mu L}{1-0.63(h/w)}\cdot \frac{1}{h^{3}w}$$

where *L*, *h* and *w* are the length, height and width of the microchannel. For the sake of simplicity, *R_i_, L_i_, h_i_, w_i_*, represent the resistance, the length, the height and the width of the *i*-channel, respectively, where *i* can be *1*, *2*, *3*, *4*, for the channels, in correspondence with the four elements, or *a*, *b*, *c* for the channels that connect them, as shown in Figure 3(b) (a symmetric microfluidic network has been designed to allow inversion of inlet and outlet as desired). Using Kirchhoff's circuit laws, we found that the condition of equal flows *Q* (*Q_1_ = Q_2_ = Q_3_ = Q_4_*) is satisfied when *R_c_ = 3R_a_*. The resistances of the channels *a*, *b* and *c* have been tuned only by changing the width *w*: in this way we avoid a superfluous increase of the microfluidic network complexity. The *1*, *2*, *3*, and *4* channel dimensions were taken equal and the same of the fist layout proposed (*w* = 250 μm and *h* = 10 μm). The previous relation thus becomes:

(10) $$w_{a}={3w}_{c}-2\cdot 0.63h$$

for the widths of channels *a* and *c*. No condition must be imposed on *w_b_*, so we have chosen a mean value between *w_a_* and *w_c_*. The design, based on Equations (9) and (10), of a microfluidic circuit which can homogeneously distribute the biomolecules in each active area, has been tested by numerically solving the incompressible fluid flow using 3D Navier Stokes equations. The model has been verified assuming *w_c_* = 100 μm (then *w_a_* = 287.4 μm, *w_b_* = 193.7 μm), and *h* = 10 μm; a surface map, giving the velocity field illustrated in Figure 4(a).

**Figure 5.**
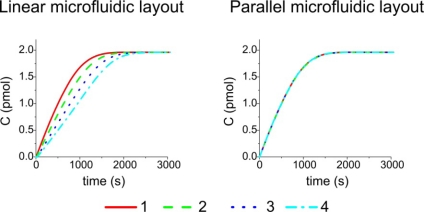
Comparison of C formation binding kinetics obtained by simulating a dynamic incubation from the first to the last element in linear and parallel microfluidic system, respectively.

From these calculations, it results that the four current flows are equal within a confidence range of less than 5%. The electrical network analogy can be thus used for the fabrication of a compact microfluidic circuit which feeds the chemical substance B in parallel. The comparison between the binding kinetics of formation of C in the four active areas in the case of the two microfluidic layouts for u_0_ = 1 mm/s is presented in Figure 4(b). From Figure 4(b), where the binding kinetic using a static flow condition is presented, it is clearly evident that the parallel microfluidic layout, which assures a homogeneous velocity field across each active area, also give rise to an equal C complex formation in all the sensing elements. The advantages of a parallel configuration are also evident in case of dynamic flow conditions. We have simulated the microfluidic circuit behavior quantifying the formation of complex C in the four active zones for a 1 mm/s solution flow. The results are reported in Figure 5.

**Figure 6.**
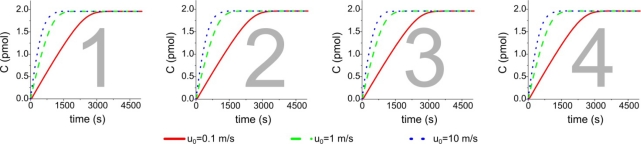
Comparison of the formation of complex compound C simulating a dynamic incubation from the first to the last element in a parallel microfluidic system for different inlet velocity values.

In a dynamic regime, the four active areas reach the same amount of C are different time points, and the saturation condition is obtained with a time difference of 48% between the last element (2,040 s) with the respect to the first element (1,380 s). By parallel microfluidics it is possible to almost cancel this delay: all the elements saturate in the same interval (the time delay is less than 1%). We have also investigated how the binding kinetics under dynamic flow conditions depend on the inlet velocity in the case of a parallel microfluidic system; the results are shown in Figure 6.

The simulations have confirmed that there are no substantial differences among the four elements in this case, and also a substantial decrease of saturation time can be noted upon increasing the inlet velocity: the saturation value is reached in 2,880 s for u_0_ = 0.1 mm/s, in 1,380 s for u_0_ = 1 mm/s, and in 1,020 s for u_0_ = 10 mm/s. We can thus conclude that the inlet velocity plays a fundamental role in the optimization of the microfluidic microarray both for static and dynamic regimes.

## 4. Conclusions

We have analysed the binding kinetics of the formation of a complex C in the case of a generic molecular interaction which could happen in the channel of a pressure driven microfluidic circuit used to assist and enhance the performances of a microarray. We have found the conditions required to optimize the uniformity of the chemical species distribution on the sensing area. Different microfluidic layouts have been proposed to improve the sensing performance. The dynamic flow condition approach seems to be the best in terms of homogeneity and time parameters for the microfluidic biosensor, but the static approach can be useful in case where very low sample consumption is necessary.
